# Towards Post-Meiotic Sperm Production: Genetic Insight into Human Infertility from Mouse Models

**DOI:** 10.7150/ijbs.60384

**Published:** 2021-06-16

**Authors:** Muhammad Azhar, Saba Altaf, Islam Uddin, Jinbao Cheng, Limin Wu, Xianhong Tong, Weibing Qin, Jianqiang Bao

**Affiliations:** 1 Division of Life Sciences and Medicine, The First Affiliated Hospital of USTC, University of Science and Technology of China, Anhui, China.; 2 The 901th hospital of Joint logistics support Force of PLA, Anhui, China.; 3 NHC Key Laboratory of Male Reproduction and Genetics, Family Planning Research Institute of Guangdong Province, China.

**Keywords:** Spermiogenesis, spermatogenesis, infertility, genetically engineered mouse model (GEMM), oligoasthenoteratozoospermia (OAT)

## Abstract

Declined quality and quantity of sperm is currently the major cause of patients suffering from infertility. Male germ cell development is spatiotemporally regulated throughout the whole developmental process. While it has been known that exogenous factors, such as environmental exposure, diet and lifestyle, et al, play causative roles in male infertility, recent progress has revealed abundant genetic mutations tightly associated with defective male germline development. In mammals, male germ cells undergo dramatic morphological change (i.e., nuclear condensation) and chromatin remodeling during post-meiotic haploid germline development, a process termed spermiogenesis; However, the molecular machinery players and functional mechanisms have yet to be identified. To date, accumulated evidence suggests that disruption in any step of haploid germline development is likely manifested as fertility issues with low sperm count, poor sperm motility, aberrant sperm morphology or combined. With the continually declined cost of next-generation sequencing and recent progress of CRISPR/Cas9 technology, growing studies have revealed a vast number of disease-causing genetic variants associated with spermiogenic defects in both mice and humans, along with mechanistic insights partially attained and validated through genetically engineered mouse models (GEMMs). In this review, we mainly summarize genes that are functional at post-meiotic stage. Identification and characterization of deleterious genetic variants should aid in our understanding of germline development, and thereby further improve the diagnosis and treatment of male infertility.

## 1. Introduction

Infertility is a rapidly rising concern among couples at reproductive age worldwide. Currently, around 6% of the married population among 15-44 years of age suffer from infertility 1. It has thus become a severe health problem that elicits many social consequences. Almost 50% of fertility-related problems are caused by male factors 2, such as Y-chromosome microdeletions, Klinefelter syndrome, obstruction and genetic abnormalities 3. In the clinics, some abnormalities can easily be screened by common cytogenetic and biochemical techniques available in most reproductive centers. However, nearly half of cases of male infertility are idiopathic and the genetic variants responsible for the compromised male fertility remain elusive.

In mammals, testis is mainly composed of tortuous, elongated seminiferous tubules in which spermatogenesis is taking place along the well-organized epithelia 4. In general, the production of competent sperm after birth in mammals necessitates three successive processes: (*i*) mitotic proliferation of spermatogonial stem cells (SSCs) and subsequent differentiation into advanced spermatogonia (differentiated spermatogonia); (*ii*) meiotic division: one-time duplication of chromosomes followed by two times of cell division; (*iii*) spermiogenesis, known as haploid spermatid development, is a process whereby the round spermatids morphologically transform into nuclei-condensed spermatozoa. These normal-looking sperm must be released from seminiferous tubules and transit through further convoluted epididymis to undergo functional maturation in order to be fertilization-competent 5.

At haploid stage, probably the most characteristic feature that distinguishes spermatid development from other somatic cell differentiation is the unique cellular structures formed, such as the acrosome and sperm tail 6. At the molecular level, spermatid chromatin undergoes drastic chromatin remodeling and genome-wide histone removal followed by protamine deposition, a process termed as histone-to-protamine transition, leading to the production of highly condensed sperm heads with much less cytosolic contents. It has thus been conventionally thought that sperm function as a “carrier” that solely delivers the paternal genetic DNA into oocyte which initiates fertilization 7. Recent progress in the field argued that the rich non-coding RNA molecules buried inside the sperm nucleus execute a significant impact on the subsequent development in the offspring epigenetically (i.e., independent of the DNA sequence) 8.

Therefore, understanding the mechanisms by which gene expression is specifically regulated to facilitate the spermatid elongation and genome-wide re-organization prepared for the sperm-egg fertilization, has significant implications for reproduction and human health. In the past, a network of genes, either germline-specific or non-specific, has been identified and characterized to be essential for male fertility based on high-throughput sequencing in the patient's pedigree or GEMMs. However, it must be pointed out that, although the testicular structure and germline developmental process resemble each other to some extent between mice and humans, there are considerable differences in both species. For example, the meiosis persists for ~25 days in men but only ~14 days in male mice. Following meiosis, the haploid spermatid differentiation takes ~16 days in men but is shorter (~9 days) in mice. Thus, we must be cautious when interpreting the causative factors and mechanisms underlying human infertility by taking advantage of the findings from mouse studies 9. In this review, we will particularly summarize those genes that are functionally important for post-meiotic male germ cell development in mice, which will aim in our understanding of human fertility.

## 2. Key phases during stepwise spermiogenesis

**Figure 1:**
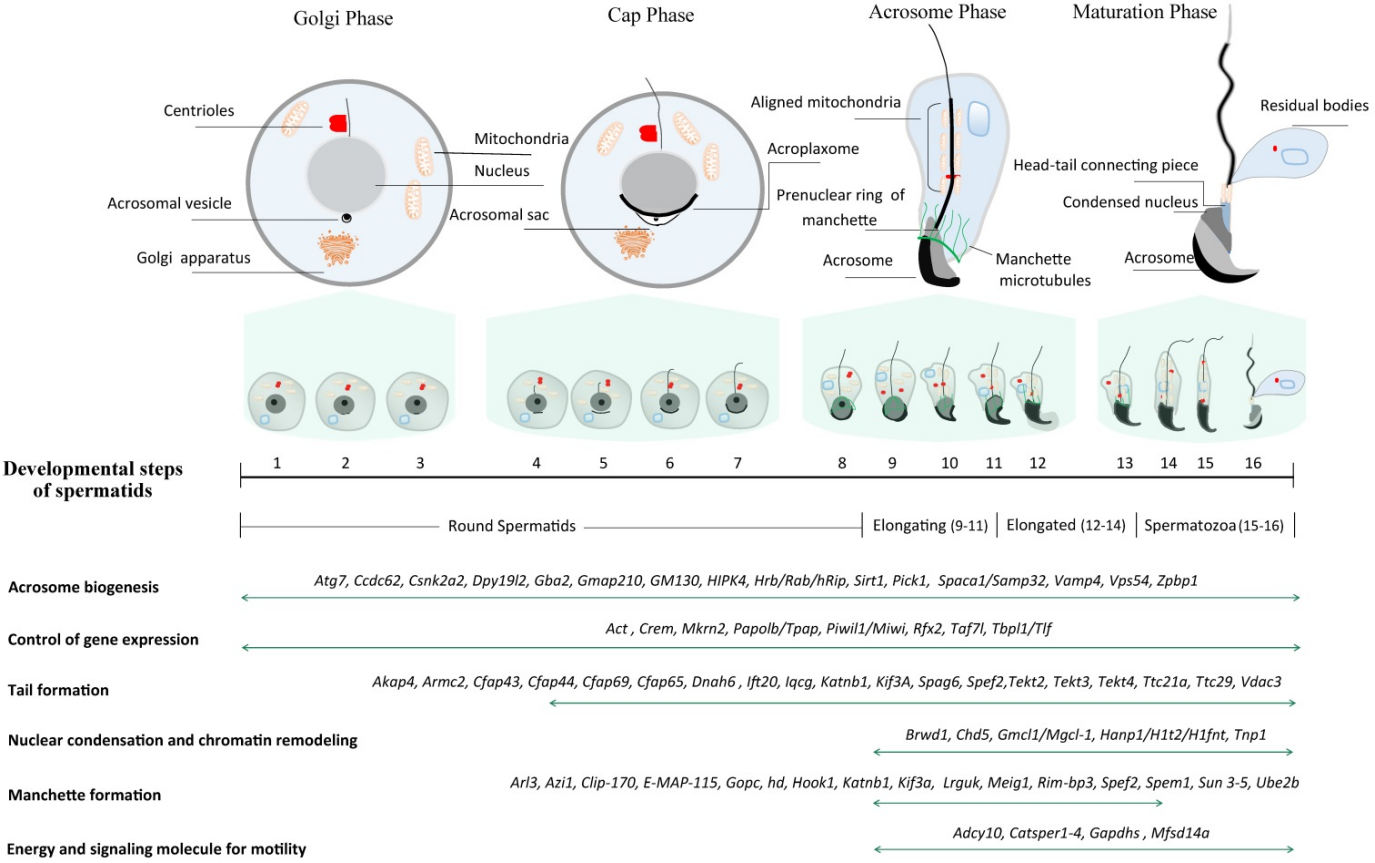
** Schematic diagram illustrating a total of 16 steps of haploid germline development in mice***.* Spermiogenesis is categorized into four phases (Golgi phase, Cap phase, Acrosome phase and Maturation phase) according to the acrosome morphology. Conventionally, spermatids are divided into 16 steps on basis of acrosome and head morphology, and the criteria is commonly leveraged to pinpoint the specific step of spermiogenic arrest through H&E staining of the sections from GEMMs. In literature, spermatids are often classified into four groups based on nuclear morphology: round spermatids (Steps 1~8), elongating/condensing spermatids (Steps 9~11), elongated/condensed spermatids (Step 12~14) and spermatozoa (Step 15~16). Representative genes essential for spermatid development are listed at the bottom.

In mice, spermiogenesis entails the dramatic morphological transformation of round spermatids into hook-shaped mature spermatozoa. Unlike somatic cells, the motile sperm are capable of fertilizing an egg through their highly specialized cell structures 10. Three major morphological changes take place in the process of spermiogenesis, including acrosome biogenesis, head reshaping, and development of a flagellum (Fig. 1). Right after meiosis, pairs of individual round spermatid are connected through intercellular bridges (Cytoplasmic bridges) through which haploid gene products transit and are shared across the cell membrane. On basis of the acrosome morphology, it can be divided into four phases during haploid spermatid development (Fig. 1) 11. The first phase is called Golgi phase in which the proacrosomal vesicles fuse to form a single large acrosomal vacuole, which contains the granules in contact with the nuclear envelope (Step 1-3). Soon after meiosis, Golgi-derived vesicles traffick through microtubules and F-actin microfilaments, and subsequently aggregate and fuse to form the acrosome sac. Round spermatids consist of a centrosome with two centrioles - one is named distal centriole (mother centriole) based on the distal location while the other is called proximal centriole (daughter centriole). During sperm flagellar formation, the centrioles undergo dynamic remodeling and serve as the nucleators of the cilia axoneme, of which the process is explicitly reviewed elsewhere 12. Upon occurrence of acrosomal sac, the centriolar pair migrates to the other pole opposite to the acrosomal vesicle wherein the axoneme starts to assemble. These distal centrioles will develop into flagellum while the proximal centrioles and pericentriolar matrix will eventually develop into head-tail coupling apparatus, which connects the sperm head and tail axoneme. In the second phase**,** also known as cap phase (Step 4-7), both the tail axoneme and acrosomal sac continue to grow. Meanwhile, the growing acrosomal sac becomes flatten and forms a cap tightly adhered to the anterior nuclear envelope through the F-actin-containing acroplaxome. The acrosome-acroplaxome complex descends caudally to the tail assembly direction in concert with the elongation of the nucleus. This process is followed by the acrosomal phase (phase 3), the acroplaxome attached to the acrosome continues to descend along the axoneme direction and spreads over the anterior nuclear envelope, in accompany with the developing of microtubule-containing ring-like manchette and the aligning of mitochondrion to the axoneme (Step 8-12). The outer dense fibers (ODFs) start to assemble and align along the developing axoneme. Upon completion of nuclear elongation, the acrosomes enter the maturation phase (phase 4) (Step 13-16) 13 when the manchette starts to dissemble and the majority of histones are step-wisely replaced by protamines whereby the chromatin condensation continues further. This process necessitates genome-wide chromatin remodeling as well as massive single-stranded breaks (SSBs) and/or double-stranded breaks (DSBs) 14.

Currently, little is known about how the histones are globally substituted and how the SSBs/DSBs are repaired in such a narrow time window, although much knowledge has gained from GEMMs that the defect in any of these steps is closely linked to the compromised male fertility. In addition, it is noteworthy to point out that the acrosomal development unique to germ cells is successive and inherently connected to nuclear elongation and condensation. Secondly, since there is only one copy of allele owing to the haploid genome *per se*, spermatids are thus not capable of repairing the DSBs via homology-directed repair (HDR) mechanism. It is, therefore, conceivable that any disturbance at this stage would not only cause decreased fertility but also induce pathogenic mutations that can be carried over to progeny 15. Finally, we must bear in mind that, although the sperm released into the lumen in the testicular tubules is morphologically identical to mature sperm, they must undergo further functional maturation during transit in the lengthy epididymis wherein the sperm might exchange information with extracellular vesicles in the fluid, in order to be capacitation-competent 16.

## 3. Morphological defects taking place during spermiogenesis

### 3.1. Acrosome assembly

As described above, the acrosome is a unique glycoprotein-rich organelle derived from Golgi complex, and thus can be readily visualized by the Periodic acid-Schiff (PAS) and PNA (Peanut agglutination assay) staining, or by electron microscopy 17. The acrosome is also enriched in protease enzymes, such as acrosin, esterases, acid phosphatases, aryl sulfatases, and glycohydrolases, Sp56/AM67, SP-10, and Hyal-PH20, which are essential for sperm-oocyte fertilization. The acrosin and SP-10 are associated with the acrosomal matrix and released after the acrosomal reaction in large part 13. SP56 is localized to the acrosomal matrix and has a specific affinity for mouse Zona pellucida sperm-binding protein 3 18. To date, Hyal-PH20 is the only ideal acrosomal membrane protein. It is a glycosylphosphatidylinositol-anchored protein and localized to both the inner acrosomal membrane and plasma membrane of sperm head. Interestingly, it has been shown to play in sperm-egg interaction that is unrelated to its enzymatic activity 19.

The sperm head shaping is an important event of spermiogenesis and is coordinated by nuclear condensation (Fig. 1). Two distinct subcellular structures are involved in the sculpting of the head into species-specific morphology (e.g., hook-like shape in mice, and oval shape in humans) -acroplaxome and manchette 20. The anchoring of the acrosome to the nuclear surface is mediated via the acroplaxome, also named prenuclear theca, which is a thin layer of cytoskeletal elements between the nuclear and acrosomal membranes. In 2002, phospholipase C zeta (PLCζ) has been discovered 21, residing in the post-acrosomal sheath of perinuclear theca and is essential for oocyte activation 22. The acroplaxome is comprised of myosin, F-actin and keratin-containing components essential for acrosome development. The acroplaxome provides mechanical support for transmitting forces around the anterior nucleus to facilitate the elongation of sperm head 23. The presence of keratin-5 and F-actin is important in the acroplaxome for force generation and transfer 24. On the other hand, it has been reported that some inhibitors related to microtubule dynamics interfere with the developing acrosomal vesicles resulting in abnormal spreading of acrosome over the nuclear membrane 25. As a result, spermatozoa carrying defective acrosomes are not capable of penetrating zona pellucida of oocytes, leading to fertilization failure 26.

### 3.2. Manchette assembly

The second key structure that is essential for sperm head shaping is manchette, which is a transient skirt-like microtubular structure surrounding the posterior nucleus present in elongating spermatids (Fig. 1). It is composed of up to 1000 microtubules and consists of α and β tubulin heterodimers. The timing of manchette appearance is strictly step dependent. The short microtubules appear first around step 7 and then assemble at step 8 27. The microtubules are closely wrapping around posterior nuclear surface till step 14 when the manchette begins disassembling 28. Through intra-manchette transport, it is thought to assist in the nucleocytoplasmic exchange, mechanical reshaping and elongation of sperm nucleus, as well as assembly of sperm tail. As a result, the excess cytosol is removed, and the nucleus becomes highly condensed. Any interference with the manchette structure or function can potentially give rise to the abnormal sperm head shape. Till now, a dozen of genes have been identified and are associated with manchette formation and development, which have been explicitly reviewed elsewhere 29. Likewise, many mutagenic agents including inhibitors of microtubule dynamics, e.g., alkylating agent, can also induce manchette malfunction. Genetic deletion or mutation studies unveiled the aberrant manchette morphology, abnormal nuclear condensation/elongation and impaired fertility in those manchette-related gene mutation mouse models 30-32.

### 3.3. Flagellar assembly

**Figure 2:**
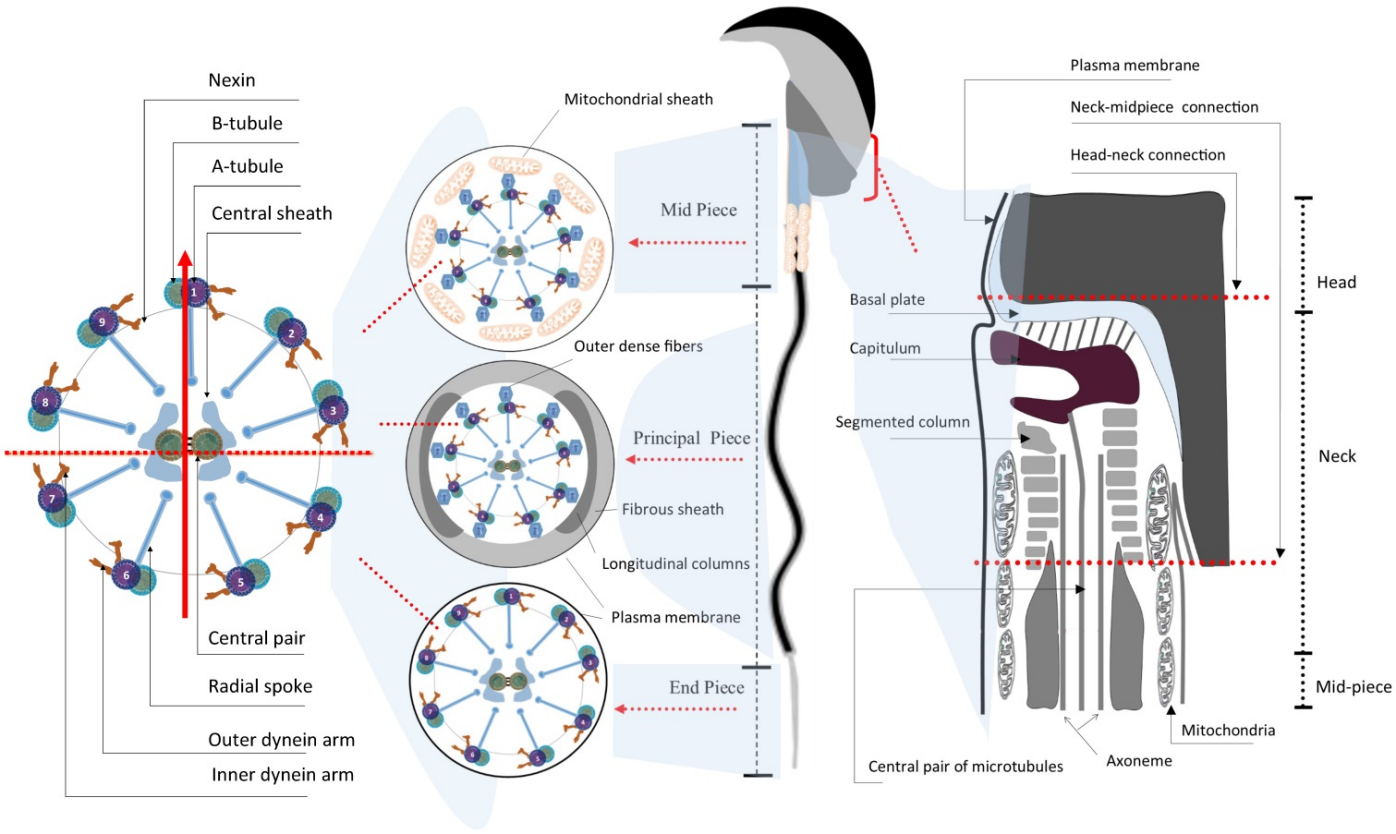
Schematic illustration of the ultrastructure of mature mouse sperm. Sperm flagellum is structurally divided into three parts - midpiece, principal piece, and end piece; Each part comprises the core axoneme, which is composed of the canonical “9+2” arrangement of microtubules, in the center. The vertical arrow points to the #1 pair of microtubules while the horizontal dash line parallels the central pair of microtubules, in the cross mark. The mitochondrial sheath and the outer dense fibers (ODFs) wrap around the mid-piece and principal piece, respectively. The connection part between sperm head and tail is the neck, also termed head-tail coupling apparatus or connecting piece (right panel) 34, 150.

The most distinctive feature of spermatids probably lies in the formation of a highly structured flagellum - the sperm tail, which is tightly relevant to sperm motility (Fig. 1 and 2) 10. Morphologically, the flagellum is highly conserved in its ultrastructure and can be divided into four parts: *i*) neck, *ii*) midpiece,* iii*) principal piece and* iv*) end piece (Fig. 2). The neck is also known as connecting piece or head-tail coupling apparatus, which anchors the tail to the head tightly through the capitulum and the segmented column as magnified under transmission electronic microscopy (TEM). This ultrastructure has been elaborately discussed by several review papers elsewhere 30, 33. The midpiece is comprised of mitochondria and 9 ODFs, which surround the 9+2 axoneme. Two ODFs are replaced in the principal piece by the longitudinal columns of the fibrous sheath (FS) connected by transverse ribs. The end piece contains only the axoneme surrounded by plasma membrane 34. Upon completion of nuclear elongation and condensation, the fully developed sperm migrate close to the lumen of seminiferous tubules and are ready to be released into the lumen in a process called spermiation 10.

As the acrosome begins to form, the pair of centrioles move to the opposite pole of acrosome to initiate axoneme formation (Fig. 1). The axoneme arises from the centrioles and extends out into the cytoplasm 24. The axoneme is comprised of characteristic “9+2” microtubules wherein a central pair of microtubules are wrapped by nine doublet microtubules. Notably, these microtubules are post-translationally modified including tyrosinated, acylated, and polyglutamylated modification 30, 33. The outer doublets contain dynein motor arms that generate forces to endow waveform motion of the flagellum. Defects in post-translational modifications of tubulins or perturbations in microtubular arrangement can impair axoneme assembly, which can be visualized by staining acetylated tubulin or by electron microscopy 30, 33. At the last step, prior to fertilization, the sperm must acquire its fertilizing capability in a process known as capacitation during transit in the female reproductive tract. Capacitation is linked to hyperactive motility acquisition, characterized by an increase in the amplitude and asymmetry of the flagellar bending, and genetic mutations causing defective capacitation are linked to male infertility 35.

Until now, whereas it remains challenging to decipher which signaling pathways are involved in the axoneme assembly and elongation *in vivo*, growing studies have discovered a large number of genes in humans with the mechanistic and phenotypic studies further characterized in mouse models, showing they are tightly associated with axoneme formation. Most often, mutations or disruptions to those genes can induce a severe type of morphological abnormality commonly observed in the clinics - multiple morphological abnormalities in flagella (MMAF), leading to male sterility, which has been elegantly summarized and reviewed in details 36.

## 4. Spermiogenic defects: Insight view from mouse models

Post-meiotic spermatids develop clonally in many species ranging from fruit flies to humans and remain connected to one another via intercellular bridges. These bridges work as a channel for sharing protein products or even small subcellular organelles such as chromatid body (CB) and mitochondria 37. In this way, the bridges enable haploid spermatids to be functionally diploid 38. Exogenous interruption or genetic mutations often result in aberrant spermiogenesis, and consequently, lead to the reduction of sperm number or motility, or morphologically or functionally defective sperm. On basis of these varied abnormalities, the most common phenotypes with clinical outcome can be generally categorized into four groups as discussed below.

### 4.1. Oligozoospermia

Oligozoospermia is a common type of human infertility, which refers to the low sperm count in the semen. According to the WHO 2010 criteria, the average sperm count is more than 60 million per ml, and a count of less than 15 million per ml is considered oligozoospermia 39. It is further sub-categorized into three classes: mild oligozoospermia (10-15 million/ml), moderate oligozoospermia (5-10 million sperm/ml), severe oligozoospermia (< 5 million sperm/ml) 40. In addition, it is now known that the disturbance in the post-meiotic spermatid development will likely cause aberrant spermatid apoptosis or premature spermatid sloughing off the seminiferous tubular epithelia, resulting in the reduction of spermatid number and hence impaired male fertility. This syndrome is more often connected to teratozoospermia and asthenozoospermia.

Until now, few genes are reported to be responsible for solely low sperm count. Fibroblast growth factors (FGFs) family contains 22 known members 41, which are involved in important cellular processes such as differentiation, migration, mitogenesis, and cell survival 42. FGF action is mediated through high-affinity binding to tyrosine kinase receptors, which include FGF receptor 1 to FGF receptor 5 (FGFR1-FGFR5) 43, 44. The role of Fgfr1 in male infertility and spermiogenesis has been assessed by dominant-negative transgenic mouse models, showing that Fgfr1 contributes to sperm production and function as the sperm daily-out has been examined low 45. Recently, whole-exome sequencing (WES) efforts revealed a handful of genetic variants that are potentially linked to human oligozoospermia, such as *HAUS7* 46, *M1AP* 47, *MAGEB4* 48, *RPL10L* 49, and *ZMYND15* 50 (Table 1).

### 4.2. Asthenozoospermia

Spermatozoa own highly specialized structures, which discriminate them from other somatic cells, especially the long tails, which are required for sperm motility. Defective microtubular assembly of sperm flagellum or functional suppression directly dampen sperm motility and thus cause reduced male fertility known as asthenozoospermia. It is one of the common types of male infertility in the clinics 51. Mouse model studies have shown that the structural integrity of the flagella is of extreme importance for normal sperm motility, and defects in the axoneme assembly render poor sperm motility 52. Not surprisingly, WES has identified a large number of genetic variants relevant to asthenozoospermia, while each variant is likely responsible for just a small fraction of patients, particularly including *ADCY10* 53, *CATSPER1-4* 54, *EIF4G1* 55, *GALNTL5* 56, *NSUN7* 57, *PLA2G6* 54, *SPAG17* 58, and *TRPC5* 59 (Table 1).

As aforementioned, the functional unit of flagella is the axoneme, which consists of structural microtubules and motor proteins that cooperate to produce waveforms and generate progressive motion in a synchronized and structured manner 60. Inner dynein arms (IDA) and outer dynein arms (ODA) are ATPase complexes consisting of multiple motor proteins and reflect distinctive functions in flagellar motility. Dynein axonemal heavy chain family proteins (DNAH1, DNAH2, DNAH17), IQ motif containing G (IQCG), and tektin protein family are structural constituents of axoneme. In patients with asthenozoospermia from different ethnic backgrounds, common deleterious mutations have been found in the genes encoding inner dynein complex and nexin-dynein regulatory complex. Nevertheless, those null mouse models have immotile sperm elicited by disassembly of the axoneme and consequently, are unable to fertilize eggs 61-63. Tektin is a family of filament-forming cytoskeleton proteins that comprise tubulin and dynein heterodimers in axoneme. It has been assumed that these constitutive proteins contribute to axonemal microtubular stability and structural complexity 64. In humans, five tektins (TEKT1, 2, 3, 4, and 5) are present in the flagella of spermatozoa. Disruption to these proteins led to a low level of ATP and clumsy movement of flagella 64-67.

Capacitation is a cAMP-dependent bicarbonate-induced process by which sperm develop the capacity to fertilize eggs. A key enzyme in the PKA pathway is a soluble adenylyl cyclase (sAC) in the sperm tail encoded by the *Adcy10*. Adenylate cyclase uses ATP as a substrate to generate cAMP, further catalyzed by PKA to phosphorylate downstream proteins 68. Male mice deficient in sAC are infertile owing to a complete lack of sperm motility 69. Noticeably, homozygous frameshift variants of *ADCY10* have been reported in asthenozoospermia patients 53. Another core messenger during capacitation is Ca^2+^ 70, although the nature of the source responsible for the inflow of Ca^2+^ remains uncertain. CATSPER1-4, four cation channel-like proteins, have pore-lining residues forming alpha subunits and three auxiliary subunits, and their amino acid sequences imitate those of the conventional four-repeat voltage-gated Ca^2+^ channel with a single repeat. They are sperm-specific heterotetrametric Ca^2+^ selective channel proteins located on the middle and principal piece of flagella that regulate the calcium entry into the sperm, which is critical for the activation of sperm capacitation and motility. Mouse knockout models exhibit drastically reduced sperm motility and are thus sterile only in the males. Recurrent genetic mutations were recently discovered in CATSPER family members in human patients with asthenozoospermia through Exome-sequencing, suggesting highly conservative roles of CATSPER members in sperm activation in mammals 54, 71-73.

Synchronized motion of the sperm tail involves energy production, commonly through oxidative phosphorylation, and signaling from its environment, and thus disturbances in the supply of energy and signaling transduction may also cause motility defects. It is generally speculated that oxidative phosphorylation is restricted to the midpiece harboring mitochondria, whilst glycolysis is confined to the principal piece 74, 75. Several glycolysis isozymes are present in mammalian sperm, including Gapdhs, Adcy10, and Vdac3. Glyceraldehyde 3-phosphate dehydrogenase-S (Gapds) is the only Gapdhs isozyme tightly bound to the fibrous sheath. Voltage‐dependent anion channel 3(Vdac3) is a non-specific hollow porin in the outer mitochondrial membrane of sperm flagella. ATP levels significantly dropped in the *Gapdhs*- and *Vdac3*-null mice leading to non-progressive and weak motility of sperm flagella 69, 76-78. However, for majority of patients with severe asthenozoospermia, the hereditary pathogenic variants, if present, remain largely undetermined, and further screening and functional studies are required to fully understand the pathogenesis of severe asthenozoospermia.

### 4.3. Teratozoospermia

**Figure 3:**
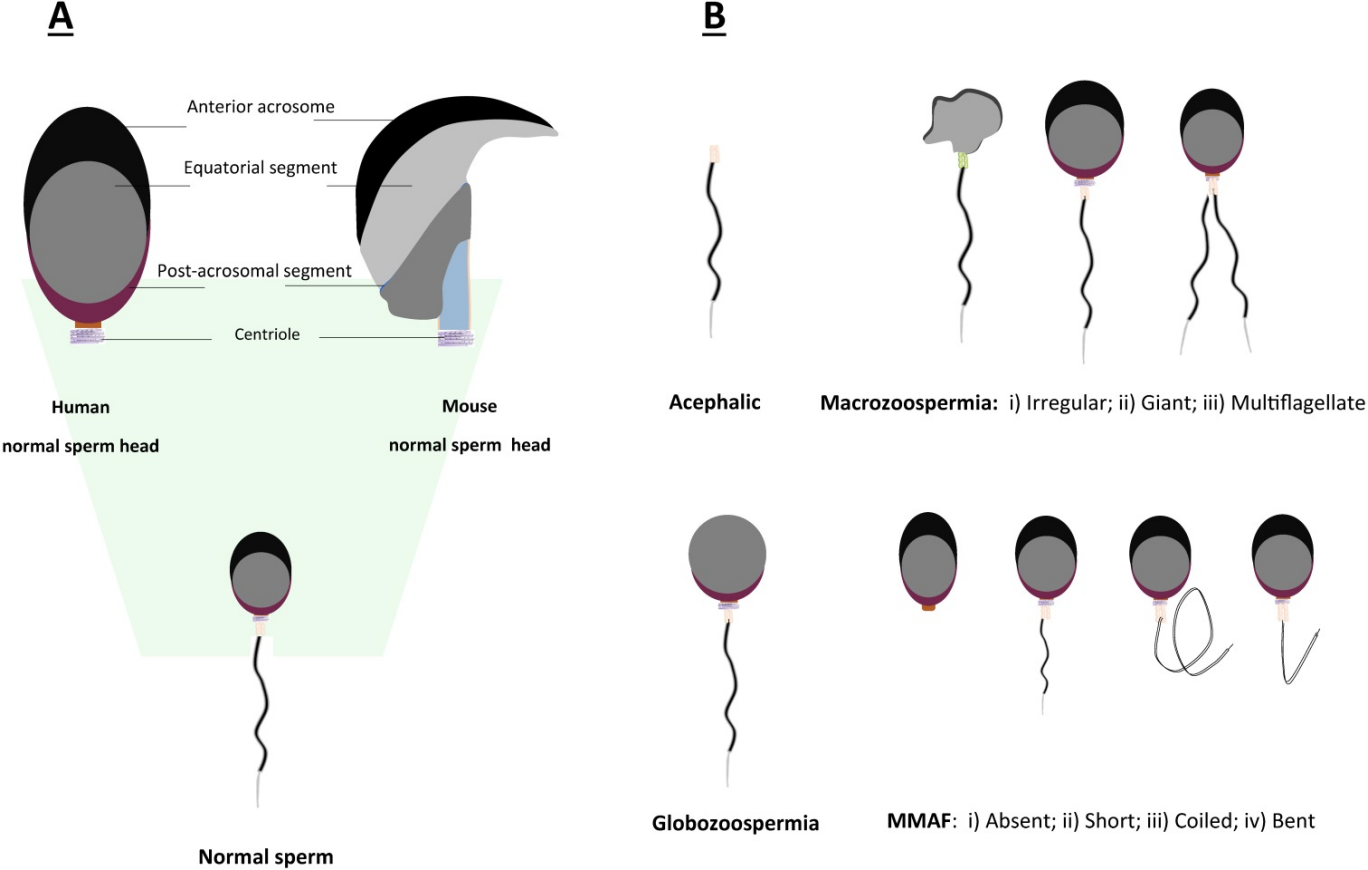
Schematic representation of morphological abnormalities of sperm in mice and humans. A. Comparison of normal sperm head morphology between mouse and human; B. Common types of aberrant morphologies of sperm in mice and humans.

Teratozoospermia is defined as a condition of male infertility characterized by the presence of abnormal morphology of sperm head or tail (Fig. 3), which is often linked to poor sperm motility. In patients with teratozoospermia, spermatozoa with irregular morphology account for more than 85% in the semen (Fig. 3). Teratozoospermia can be classified into polymorphic and monomorphic. Polymorphic teratozoospermia is thought to present an abundance of spermatozoa with heterogeneous forms of abnormalities. In contrast, monomorphic teratozoospermia exhibit a particular homogeneous morphological defect. Three well-described representative types of monomorphic teratozoospermia, i.e., macrozoospermia and globozoospermia, acephalic sperm syndrome have been reported thus far 26, 79, 80.

Nuclear elongation and chromatin condensation occur concomitantly, accompanied by the displacement of nuclear DNA-wrapped histones by protamines. Meanwhile, during this histone-to-protamine transition process, multiple histone variants are expressed, such as tH2A, tH2B, H1t, Hanp1, ssH2B, and Hils1, which are essential for sperm head maturation. Therefore, not surprisingly, genetic ablation of those histone variants usually caused severe defect in sperm condensation and male sterility 81-85. In condensing spermatids, Tnp1 is loaded onto the nucleosomes, promoting protamines deposition and processing, and removal of histone proteins. Mice lacking Tnp1/2 are sterile, presenting poor motility and irregular chromatin condensation 86, 87. Inside the sperm nucleus, the chromatin undergoes global modifications and remodeling during condensation, of which the process, however, remains largely unknown. We thus envision that further attempts to investigate the molecular changes throughout nuclear condensation will likely provide insights into the molecular mechanisms underlying patients with teratozoospermia.

#### 4.3.1. Acephalic sperm syndrome

One rare subtype but highly severe condition is decaudated sperm (acephalic sperm or headless sperm). This syndrome is most often caused by the defective formation of the head-tail coupling apparatus (Fig. 2). Acephalic spermatozoa syndrome has been reported in humans for decades and is characterized by semen often containing sperm flagella without heads 88. They are headless tails with a bulbous drop of residual cytoplasm but sometimes they are analyzed erroneously as a globozoospermia-like condition. A few acephalic spermatozoa-related genes, such as *Sun5, Pmfbp1, Spata6,* and *Spatc1l,* have been reported. Ablation of these genes in mice led to the production of acephalic sperm 89-92. Sun5 has been identified to localize on the sperm nuclear membrane facing the acrosome and to bind the acrosome, and *SUN5* mutations are most often found in patients suffering from acephalic sperm syndrome so far 93.

Teratozoospermia is commonly a severe consequence of defective flagellar assembly or destructive function of flagella. As discussed above, both intramanchette transport (IMT) and intraflagellar transport (IFT) are functionally similar to each other and both are important for the assembly and maintenance of cilia during the tail formation. By IMT, target cargo proteins are transported from nucleus to cytosol, of which the process is essential not only for nuclear condensation but also for tail assembly 94. This is exemplified by protein-coding genes involved in cargo transport, such as *Kif31, Meig1, Spef2* and *Stk33*. Deletion of *Kif31* and *Meig1* caused a substantial lethal effect on manchette formation and sperm tail production, resulting in deformed head (disrupted structure of manchette) and tail (axoneme disorganization, failure of flagellar formation) 95, 96. *Spef2* and *Stk33* KO mice exhibited a similar phenotype with the irregular formation of manchette and ultimately highly disorganized tail structure 97, 98.

#### 4.3.2. Macrozoospermia

Macrozoospermia is a very rare syndrome found in less than 1% of infertile males. It can be described as the presence of an enlarged sperm head resulting from the failure of nucleus condensation (Fig. 3). Currently, *AURKC* is the only well-known gene that manifests this kind of phenotype resulting in male infertility. Two brothers underwent genetic study of the *AURKC* gene presenting a relatively large-headed sperm phenotype. Aurkc deficient sperm of mice showed defects including heterogeneous chromatin condensation, and blunt heads with loose acrosomes 99, 100.

#### 4.3.3. Globozoospermia

Globozoospermia is a rare type of teratozoospermia that accounts for less than 0.1% of male infertility. Any disruption to the acrosome formation might result in malformation of the acrosome and the condition is characterized by the presence of a round-shaped sperm head as well as the lack of acrosome 74. The complexity of acrosome biogenesis, which lasts about two weeks in mice and one month in humans, makes it susceptible to endogenous or exogenous insults that may be detrimental for male fertility. Animal studies have unveiled a few genes and their pathways related to globozoospermia in haploid spermatids (Table 1)*,* including *Dpy19l2* 101, *Dnah6* 102, *Pick1* 103, and *Spata16* 104, that account for the genetic causes of globozoospermia. In particular, *DPY19L2* variants appear to be responsible for globozoospermia in most patients 105.

As described earlier, the fusion of Golgi-derived vesicles culminates in acrosome formation, of which the process has been tightly regulated and involves numerous vital players. Pick1 (protein interacting with C kinase 1) facilitates the vesicle trafficking from Golgi complex to acrosomes through protein trafficking. By gold particle labeling, it has been shown to localize in Golgi-related vesicles in round spermatids. Gmap210 is a Golgi membrane receptor for Ift20 and is critical for Golgi apparatus assembly 106, 107. In these gene knockout mouse models, ultrastructural analysis through TEM unveiled oblong head along with deformed acrosome, and abnormal formation of mitochondria sheath. Another gene, *Hrb,* encodes a protein related to nucleoporins essential for acrosome formation and deficient Hrb mice had round-headed spermatids and a complete lack of acrosome 108. Dpy19l2 (a transmembrane protein found in the inner nuclear membrane), Vps54 (Golgi-associated retrograde protein complex from the endosome for tethering of vesicles to the trans-Golgi network), and Mfsd14a (transmembrane protein) are responsible for the detachment of acroplaxome from the nucleus. *Dpy19l2* KO led to the intrusion of a layered structure present at the nuclear-acrosome junction, Vps54 deficient mice lack the true acrosome, and Mfsd14a-null mice failed to form an acrosome surround the nucleus 109-111.

Growing evidence showed that the autophagic molecular machinery, evolutionarily conserved from yeast to mammals, is involved in the transfer and fusion of Golgi-derived vesicles to the acrosome during acrosome biogenesis. The core members for autophagosome formation include ubiquitin-like conjugation proteins, Atg5/Atg7/Atg12 and microtubule-associated protein 1A/1B-light chain 3 (LC3) lipids. Atg7 is homologous to the ubiquitin-activating enzyme E1 essential for the conjugation structures 112, 113. *Atg7* mutant mice have aberrant round-headed spermatozoa with abnormal acrosome formation, which results from the failure of proacrosomal vesicle fusion and membrane trafficking 113, 114. In round spermatids, Gopc partially colocalized with sp56 on the acrosome but failed to localize to the acrosome in spermatids deficient in Atg7 115, 116. Atg5 is also involved in the correct formation of acrosome, and upon knockout, Atg5-null sperm exhibit disorganized mitochondrion, misshapen heads and tails 114. Interestingly, Sirt1 also appears to be involved in acrosome biogenesis via the autophagy pathway. The germline-specific *Sirt1* KO mice have round-headed sperm with aberrant acrosome 117.

#### 4.3.4. Multiple morphological abnormalities of the sperm flagella

The syndrome of multiple morphological abnormalities of the sperm flagella (MMAF) is considered as a specific subtype of asthenoteratozoospermia (Fig. 3) 52. MMAF is characterized by mosaic anomalies of flagella involving coiled, twisted, irregular, shortened, or absent flagella 118, 119. Other forms of asthenozoospermia, including MS defects, primary ciliary dyskinesia-related defects, ion channel defects and annulus instability should be distinguished from MMAF, and they are in general confined to the mid-piece that continuously displays mitochondrial ultrastructural changes or the lack of mitochondria 36. Recent studies by WES screening have discovered a myriad of genetic variants as causes of MMAF patients, such as Dynein axonemal heavy chain family proteins (*DNAH1* 120,* DNAH2* 121,* DNAH6* 102*, DNAH8* 122 *and DNAH17* 123, and Cilia and flagella associated protein family (*CFAP43* and *CFAP44* 124,* CFAP58* 125,* CFAP65* 126, *CFAP69* 127,* CFAP70* 128,* CFAP74* 63 and *CFAP251* 129). Apart from these two families, many other genes are also disclosed through WES, particularly *AK7* 130, *ARMC2* 131, *DZIP1* 132, *FSIP2* 133, *QRICH2* 134, *SPEF2* 135, *TTC21A* 136, *TTC29* 137 and *WDR66* 138 (Table 1). We expect that more other genetic variants concerning MMAF would be uncovered owing to the declining cost and advance of high throughput sequencing. The genetic mutations and featured phenotypes of mouse models for genetic variants discovered in MMAF patients have been explicitly discussed in several recent review articles 36, 139.

### 4.4. Oligoasthenoteratozoospermia

Oligoasthenoteratozoospermia (OAT) is the most common but severe form of pathogenic condition of male infertility characterized by a combination of semen abnormalities as described above, including low sperm count, poor motility, and abnormal morphology 140. Noteworthily, OAT syndrome patients have sometimes been categorized into other types of defects in the clinics. Recent studies in both mouse models and human patients have revealed abundant genetic variants that account for OAT. However, the causative genetic mutations underlying at least 50% of OAT patients remain elusive. As mentioned above, any defect taking place throughout the prolonged development of haploid spermatids is likely to elicit OAT (see Table 1).

#### 4.4.1. Intraflagellar transport (IFT)

As partially described above, numerous genes involved in intraflagellar transport relating to flagellar assembly exert vital roles during sperm head reshaping and tail formation. As a result, genetic mutations and functional disruption will cause OAT syndrome. *Katnb1* and* Lrguk-1* are predominantly expressed in germ cells and important for depolarization of microtubules during sperm head and tail axoneme development. Genetic inactivation of both genes resulted in OAT phenotype due to manchette dysfunction and tail defects 141, 142. *Ift20* and *Ift140* are localized to the Golgi complex and involved in cargo protein transport among Golgi complex, microtubules and flagella, whereby the excess residual cytosol is removed during flagellar assembly. There is no wonder that genetic disruption of Ift20 and Ift140 led to male infertility and OAT phenotype because of severe morphological defects in the sperm head and tails 143-145. Likewise, IFT27 has been shown to bind IFT25 and is essential for flagellar formation even they are not required for ciliogenesis in somatic cells 146.

#### 4.4.2. Acrosome formation

A handful of genes, such as *Vps13b, GalNAc-T3, Orp4,* and* Hipk4*, have been identified in the proper organization of Golgi apparatus and fusion of proacrosomal vesicles. Vps13b is involved in the sorting and transport of proacrosomal vesicles to the nuclear dense lamina in early spermatids. Oxysterol-binding protein (OSBP)-related protein 4 (Orp4) is a cytosolic receptor with a high affinity for oxysterols and transport cholesterol among intracellular organelles. Galnt3 encodes UDP-GalNAc polypeptide N-acetylgalactosaminyltransferase 3 that facilitates the fusion of proacrosomal vesicles. Hipk4 (homeodomain interacting protein kinase 4) functions as a critical regulator of the acrosome-acroplaxome complex. Genetic deletions of these genes all gave rise to OAT phenotype, mostly exhibiting fragmented and unfused acrosomes 147-149.

## 5. Prospects

Male germline development is a unique and sophisticated process in that it undergoes successive mitosis, meiosis and haploid differentiation. Abundant genes are spatiotemporally regulated and highly expressed in meiotic spermatocytes and round spermatids to guide correct spermatogenesis. As such, our current mechanistic understanding of male germline development is gained largely from mouse models. However, we must keep in mind that there are still limitations to using mouse models in that the germline in both species differs in many aspects, such as the different spermatogenic stem cell markers and differential chromatin condensation. In the clinics, while defective meiosis usually caused severe azoospermia, *i.e.*, absence of sperm, any disturbance during the haploid stage most often caused either severe oligozoospermia, asthenozoospermia, teratozoospermia, or a combination (OAT). Luckily, the patients suffering from these kinds of syndromes can resolve to intracytoplasmic sperm injection (ICSI) approach, which is commonly adopted in the reproductive center and has been a powerful means to overcome fertility issues. However, we must bear in mind that the sperm in those patients with decreased fertility might be genetically defective, and thus *in vitro* assisted reproductive techniques might cause the defective DNA mutations to be carried over to the offspring. Additionally, due to the lack of the long-term health record of babies born through *in vitro* fertilization (IVF) techniques, it is still not well-known or controversial if IVF techniques themselves, e.g., the culture medium, ROS exposure in culture, choice of sperm, etc, could somehow have an unexpected impact on progeny development in the future. Thus far, whole-genome screening by next-generation sequencing (e.g., exome-sequencing) combined with mouse models, have been validated as an effective way to investigate the causative roles and mechanisms of genetic variants of interest, and hence the landscape of genetic variants yet to-be-identified will undergo rapid expanding. This will lead to improved diagnosis and treatment for patients suffering sterility. In conjugation with the pre-implantation genetic testing (PGT), we envision that the transmission of more deleterious genetic mutations causing congenital diseases will be blocked from one generation to the next, further reducing the birth defects and improving human health worldwide.

## Figures and Tables

**Table 1:** Genes involved in spermiogenesis in mice and their corresponding mutation in humans.

Gene Name	Knockout Mice Phenotype	Species	Phenotype	Reference(s)
*Bromodomain and WD repeat domain containing 1 (BRWD1)*	Low sperm count, deformed spermatid heads, abnormal acrosome morphogenesis, defective chromatin condensation	Human, Mouse	OAT	151, 152
*Coactivator associated arginine methyltransferase 1 (Carm1)*	Low sperm count, poor motility, abnormal morphology includes headless sperm with a bent midpiece	Mouse	OAT	153
*Coiled-coil domain containing 62 (Ccdc62)*	Aberrant sperm phenotypes, impaired sperm motility, deformed acrosome, retention of cytoplasmic fragments on the sperm head and neck,	Mouse	OAT	154
*UDP-GalNAc: polypeptide N acetylgalactosaminyltransferase3 (Galnt3)*	Low sperm count, deformed acrosome,	Mouse	OAT	149
*Germ cell-less 1, spermatogenesis associated* *(Gmcl1/mGCL-1)*	Low sperm count with poor motility, abnormal head, blunt acrosome, round/narrowed heads, insufficient chromatin condensation, multiple heads and flagella	Mouse	OAT	155
*Homeodomain interacting protein kinase 4 (Hipk4)*	Low sperm count, poor motility, aberrant head, abnormal tail (bent and coiled), ectopic localization of acrosome components	Human, Mouse	OAT	148
*Intraflagellar transport 20 (Ift20)*	Low sperm count, poor motility, swollen head and abnormal tail (kinked tail, short tails or tailless)	Mouse	OAT	145
*Intraflagellar Transport 140 (IFT140)*	Low sperm count, poor motility, amorphous sperm heads, short/bent flagella	Human, Mouse	OAT	143, 144
*Katanin regulatory subunit B1 (KATNB1)*	Low sperm count, poor motility, abnormal manchette, knob-like head	Human, Mouse	OAT	32, 156
*Leucine-rich repeats and guanylate kinase-domain containing isoform 1 (Lrguk-1)*	Low sperm count, poor motility, misshapen heads, shortened tails, acrosome and the acroplaxome detached from the nuclear membrane in elongated spermatids, abnormal grass skirt-like manchette structure	Mouse	OAT	141
*Makorin-2 (MKRN2)*	Low sperm count, poor motility, aberrant morphology, spermiation failure, misarrangement of ectoplasmic specialization in testes	Human, Mouse	OAT	157
*Oxysterol-binding Protein (OSBP)-related Protein 4 (Orp4)*	Abnormal head, disturbed mitochondrial distribution	Mouse	OAT	147
*Septin4 (Sept4)*	Poor motility, abnormal morphology, defective annulus or bent neck, defects in mitochondrial architecture and acrosome	Mouse	OAT	158
*Septin12 (SEPT12)*	Low sperm count, poor motility, distinctive sperm morphology, sloughing of round spermatids, mitochondria with deformed acrosome	Human,Mouse	OAT	159, 160
*Serine protease inhibitor Kazal-type 2 (Spink2)*	Missing proacrosomal vesicles fusion, disarrangement of the Golgi apparatus, and bent flagellum	Mouse	OAT	161
*TATA-box binding protein associated factor 7 like (Taf7l)*	Low sperm count, poor motility, abnormal morphology, compromised fertility	Mouse	OAT	162
*Vacuolar protein sorting-associated protein (Vps13b)*	Disorganization of the Golgi apparatus, degeneration of spermatid, deformed head	Mouse	OAT	163
*H1.7 linker histone (Hanp1/H1t2/H1fnt)*	Poor motility, aberrant chromatin packaging, delayed nuclear condensation, detachment of acrosome	Mouse	OAT	164
*Serine/threonine kinase 36 (Stk36)*	Poor motility, club-shaped head with large gap between acrosome and the nucleus, absence of central pair of flagellar axonemes	Mouse	OAT	31
*Adenylate cyclase 10 (ADCY10)*	Poor and lack of progressive sperm motility, deficit in cyclic adenosine monophosphate (cAMP)	Human, Mouse	Asthenozoospermia	53, 69
*A-kinase anchoring protein 4 (AKAP4)*	Poor motility, diameter of principal piece reduced, distal flagellum splits apart into several filaments, incomplete development of fibrous sheath	Human, Mouse	Asthenozoospermia	165, 166
*Cation channel sperm associated 1-4 (CATSPER1-4)*	Hyperactivated motility failure	Human, Mouse	Asthenozoospermia	54, 71-73, 167
*Polypeptide N-Acetylgalactosaminyltransferase Like 5 (GALNTL5)*	Poor motility, lack of Acrosomal Component, ubiquitin-proteasome systems aberrant distribution	Human, Mouse	Asthenozoospermia	56, 168
*Glyceraldehyde-3-phosphate dehydrogenase, spermatogenic (GAPDHS)*	Poor motility, sluggish movement, bending of flagellar in the middle piece, declined concentration of ATP	Human, Mouse	Asthenozoospermia	76, 77
*IQ motif containing G (IQCG)*	Poor motility, missing flagella, short and disorganized structure of tail, deformed nucleus	Human, Mouse	Asthenozoospermia	62, 169
*Mouse dynein heavy chain 7 (Mdhc7)/(DNAH1)*	Poor motility, lack of progressive movement and straight-line velocity, missing FS, longer ODF 1 and 9 with asymmetric mitochondrial line, Length of midpiece increased 2-3 times	Human, Mouse	Asthenozoospermia	120, 170
*Sperm Associated Antigen 17 (SPAG17)*	Poor motility, irregular nuclear shape with detached acrosomes, altered manchette microtubules, reduced chromatin condensation	Human, Mouse	Asthenozoospermia	58, 171
*Tecktin-2 (TEKT2)*	Bent flagella, loss of the inner dynein arm structures	Human, Mouse	Asthenozoospermia	64, 66
*Tecktin-3 Tekt3*	Poor motility, defective flagellar pattern with abnormal bending between 90° and 180°, midpiece thinning	Mouse	Asthenozoospermia	67
*Tecktin-4 Tekt4*	Poor motility, defect in flagellar bending restricted to the end piece, twitching motion of principal piece, disorganized sub-mitochondrial reticulum	Mouse	Asthenozoospermia	65
*Ttransition protein 1 (TNP1)*	Poor and lack of progressive motility, DNA breaks, abnormal pattern of chromatin condensation, less condensed chromatin, large rod-like structures	Human, Mouse	Asthenozoospermia	86, 172, 173
*Voltage-dependent anion channel 3 (VDAC3)*	Poor motility, loss of one outer doublet, axonemal defects, aberrant appearance of mitochondria along the midpiece	Human, Mouse	Asthenozoospermia	78, 174
*Sperm associated antigen 6 (Spag6)*	Low sperm count, poor motility, loss of the sperm head, midpiece fragmentation, truncated flagella, lack of central pair of microtubules, alterations in the outer dense fibers and fibrous sheath	Mouse	Asthenozoospermia	175
*Aurora Kinase C (AURKC)*	Defective nuclear chromatin condensation with defective acrosome, blunted heads	Human, Mouse	Macrozoospermia	99, 100
*ADP ribosylation factor like GTPase 3 (Arl3)*	Abnormal head shape, lasso-like coiled tail	Mouse	Teratozoospermia	176
*5-azacytidine* *induced gene 1 (Azi1)*	Defective manchette structure, misorientation of elongated spermatids, most of the sperm lack flagella while the remaining axoneme truncated	Mouse	Teratozoospermia	177
*Cadherin 5 (CHD5)*	Abnormal head morphology (nuclear deformation, partial failure of chromatid condensation)	Human, Mouse	Teratozoospermia	178, 179
*CAP-GLY domain containing linker protein 170 (Clip-170)*	Abnormal hook-shape head, absence of manchette microtubules along with the nuclear envelope	Mouse	Teratozoospermia	180
*Epithelial Microtubule-Associated Protein Of 115 (E-map-115)*	Abnormal head (Nuclei deformation, microtubules of manchette reduced)	Mouse	Teratozoospermia	181
*Hypodactylous (hd)*	Detached centrosome, asymmetric and ectopic perinuclear ring of manchette, abnormal assembly of ODFs resulting in decapitated tails	Mouse	Teratozoospermia	182
*Hook microtubule tethering protein 1 (HOOK1)*	Elongated manchette with conically shaped, club-shaped nucleus, positional abnormalities of ODFs and FS result in lasso-like tail formation	Human, Mouse	Teratozoospermia	183, 184
*Kinesin family member 3A (Kif3a)*	Low sperm count, malformed head, detached perinuclear ring, abnormal manchette, absence of axonemal and flagellar structures, displaced mitochondria, microtubules, ODFs, FS	Mouse	Teratozoospermia	95
*Meiosis/Spermiogenesis Associated 1* *(Meig1)*	Disrupted microtubular organelle and flagellar formation, disrupted manchette	Mouse	Teratozoospermia	96
*Polyamine Modulated Factor 1 Binding Protein 1 (PMFBP1)*	Flagella without heads, retention of cytoplasm, abnormal axoneme assembly and misarranged mitochondria inside flagellum	Human, Mouse	Teratozoospermia	90
*RIMS binding protein 3* *(Rim-bp3)*	Severe malformation of the sperm head and deformed nucleus with enlarged perinuclear space, misplaced acrosome	Mouse	Teratozoospermia	185
*Spermatogenesis associated 6 (Spata6)*	Acephalic spermatozoa	Mouse	Teratozoospermia (acephalic sperm)	92
*spermatogenesis and centriole associated 1 like (Spatc1l)*	Acephalic spermatozoa	Mouse	Teratozoospermia (acephalic sperm)	91
*Sperm flagellar 2 (Spef2)*	Short and bent tail at all steps of spermatid elongation, absence/twin tails at steps 15-16, missing central pair of the axonemal microtubules and defective assembly of tail accessory structures	Mouse	Teratozoospermia	98
*Spermatid maturation 1 (Spem1)*	Poor motility, deformed head, completely bent tail, retained cytoplasm	Mouse	Teratozoospermia	186
*Serine/threonine kinase 33 (Stk33)*	Head-dislocation, Misarrangement of microtubules and mitochondria	Mouse	Teratozoospermia	97
*Ubiquitin conjugating enzyme E2 B (UBE2B)*	Aberrant head morphology, ectopic manchette, middle piece deformation	Human, Mouse	Teratozoospermia	187, 188
*Armadillo Repeat Containing 2 (ARMC2)*	Poor motility, short, coiled, and absent flagella, complete deficiency of motility	Human, Mouse	MMAF	131
*Cilia and Flagella Associated Protein 43-44 (CFAP43-44)*	Poor motility, disorganization in fibrous sheaths and periaxonemal structures, distorted cytoskeletal components, lack of central microtubules with disorganized ODFs	Human, Mouse	MMAF	124, 189
*Cilia and Flagella Associated Protein 65 (CFAP65)*	Lack of progressive motility, absent or short flagella, lack of central microtubules with disorganized ODFs	Human, Mouse	MMAF	126, 190
*Cilia and Flagella Associated Protein 69 (CFAP69)*	Shortening of midpiece and principal piece, shorter flagella, abnormal head morphology	Human, Mouse	MMAF	127
*Dynein Axonemal Heavy Chain 17 (DNAH17)*	Impaired sperm flagellar assembly with disorganized axonemal structure	Human, Mouse	MMAF	123
*Glutamine Rich 2 (QRICH2)*	Sperm flagella with short, bent, coiled and other irregular shapes, displacement of the central microtubules and ODF, abnormal 9 + 2 structure	Human, Mouse	MMAF	119, 134
*Tetratricopeptide Repeat Domain 21A (TTC21A)*	Poor motility, aberrant structure of connecting piece, lack of central microtubules with disorganized ODFs, extra peripheral microtubule doublets, absent dynein arms	Human, Mouse	MMAF	136
*Tetratricopeptide Repeat Domain 29 (TTC29)*	Poor motility (lower progressive motility), axonemal disorganization, lack of central microtubules with disorganized ODFs,	Human, Mouse	MMAF	137
*Autophagy related 7 (Atg7)*	Round-headed sperm, lack of acrosome	Mouse	Globozoospermia	116
*Casein kinase 2 alpha 2 (Csnk2a2)*	Abnormal head with bent flagella, deformed acrosome that detached from the nucleus	Mouse	Globozoospermia/Oligozoospermia	191
*Dpy-19 like 2 (DPY19L2)*	Round-headed sperm, midpiece defect, lack/deformed acrosome, 70% show absence of mitochondria while the remaining have a disorganized sheath, 20% have coiled tail	Human, Mouse	Globozoospermia	105, 111
*Glucosylceramidase beta 2 (Gba2)*	Low sperm count, poor motility, larger and round-headed acrosome, disordered mitochondria, condensed pyknotic nuclei	Mouse	Globozoospermia	192
*Golgi matrix protein 130 kD (Gm130)*	Lack of acrosome, round-headed sperm, aberrant arrangement of mitochondria	Mouse	Globozoospermia	193
*Golgi-associated microtubule-binding protein 210 (Gmap210)*	Low sperm count, deformed acrosome, abnormal mitochondria sheath, dispersed microtubules and ODFs in the cytoplasm, ectopic perinuclear ring and elongated manchette	Mouse	Globozoospermia	107
*Golgi associated PDZ and coiled-coil motif containing (Gopc)*	Poor motility, round-headed, coiled tail, fragmented acrosome and disarrangement of mitochondria, some acrosomes completely lost	Mouse	Globozoospermia	115
*Hrb (Rab or* *hRip)*	Round-headed spermatids, lack of acrosome due to failure of proacrosomic vesicles to fuse	Mouse	Globozoospermia	108
*Heat shock protein 90 beta family member 1 (Hsp90b1)*	Poor motility, large round-headed sperm, lack/abnormal acrosome, disorganization of mitochondria, high amount of cytoplasm around the nucleus	Mouse	Globozoospermia	194
*Major facilitator superfamily domain containing 14A (Mfsd14a)*	Low sperm count, round-headed spermatids, deformed head with anomalous nuclear condensation, failure of acrosome formation	Mouse	Globozoospermia	109
*Protein interacting with* *(PICK1)*	Low sperm count, poor motility, abnormal head (irregular shape and malformation of acrosome with mislocalization)	Human, Mouse	Globozoospermia	103, 195
*Sirtuin 1 (Sirt1)*	Low sperm count, round-headed spermatozoa, Abnormal acrosome biogenesis, disrupted autophagic flux	Mouse	Globozoospermia	117
*Sperm acrosome associated 1 (SPACA1/SAMP32)*	Round-headed spermatozoa, small acrosomes of deformed shape, aberrant assembly of mitochondria inside large cytoplasmic droplets, ectopic localization of nuclei and flagella	Human, Mouse	Globozoospermia	196
*VPS54 subunit of GARP complex* *(Vps54)*	Defective cytoskeletal structure and head, failure of acrosome to attach to the nucleus, irregularity of mid-piece and tail	Mouse	Globozoospermia	110
*Sad1 And UNC84 Domain Containing 3 (Sun3)*	Low sperm count with missing/mislocalized/fragmented acrosome, absence of manchette microtubules	Mouse	Globozoospermia-like	197
*Sad1 and UNC84 domain containing 4 (SUN4)*	Aberrant roundish spermatids, severely impaired head, spermatozoa encircled by their tail, coiled tail, disorganized manchette	Human, Mouse	Globozoospermia-like	29, 198
*Sad1 And UNC84 Domain Containing 5 (SUN5)*	Acephalic spermatozoa	Human, Mouse	Globozoospermia-like (acephalic sperm)	89, 93
*Zona pellucida binding protein (ZPBP1)*	Poor motility, round-headed sperm, coiled tail around the nucleus, lack of acrosome, disorganized mitochondria, and detached flagella	Human, Mouse	Globozoospermia	199, 200
*Fibroblast Growth Factor Receptor1 (Fgfr-1)*	Low sperm count, impaired sperm capacitation	Mouse	Oligozoospermia	45
